# Chromosome-level genome assembly of scalloped spiny lobster *Panulirus homarus homarus*

**DOI:** 10.1038/s41597-025-05253-9

**Published:** 2025-05-28

**Authors:** Dongfang Sun, Jianjian Lv, Baoquan Gao, Shaoting Jia, Ping Liu, Jian Li, Jitao Li, Xianyun Ren

**Affiliations:** 1 National Key Laboratory of Mariculture Biobreeding and Sustainable Goods, Yellow Sea Fisheries Research Institute, Chinese Academy of Fishery Sciences, Qingdao, Shandong 266071 China; 2 Laboratory for Marine Fisheries Science and Food Production Processes, Laoshan Laboratory, Qingdao, Shandong 266237 China

**Keywords:** Biooceanography, Marine biology

## Abstract

Lobsters, aquatic organisms of significant economic value, hold an important position in the global aquaculture and fisheries industries. However, due to overfishing and ecological change, the populations of certain lobster species have declined dramatically, prompting conservation efforts in various countries. However, limited genomics research has restricted our capacity to conserve and exploit lobster germplasm resources. Here, we present a chromosome-level reference genome for *Panulirus homarus homarus* constructed using PacBio long-read sequencing and Hi-C data. The genome assembly size was 2.61 Gb, with a contig N50 of 5.43 Mb, and a scaffold N50 of 36.69 Mb. The assembled sequences were anchored to 73 chromosomes, covering 96.05% of the total genome. A total of 25,580 protein-coding genes were predicted, and 99.98% of the genes were functionally annotated using various protein databases. The high-quality genome assembly provides a valuable resource for studying the biology and evolutionary history of *P. h. homarus*, and could facilitate sustainable resource management, aquaculture, and conservation of the species.

## Background & Summary

**Fig. 1:**
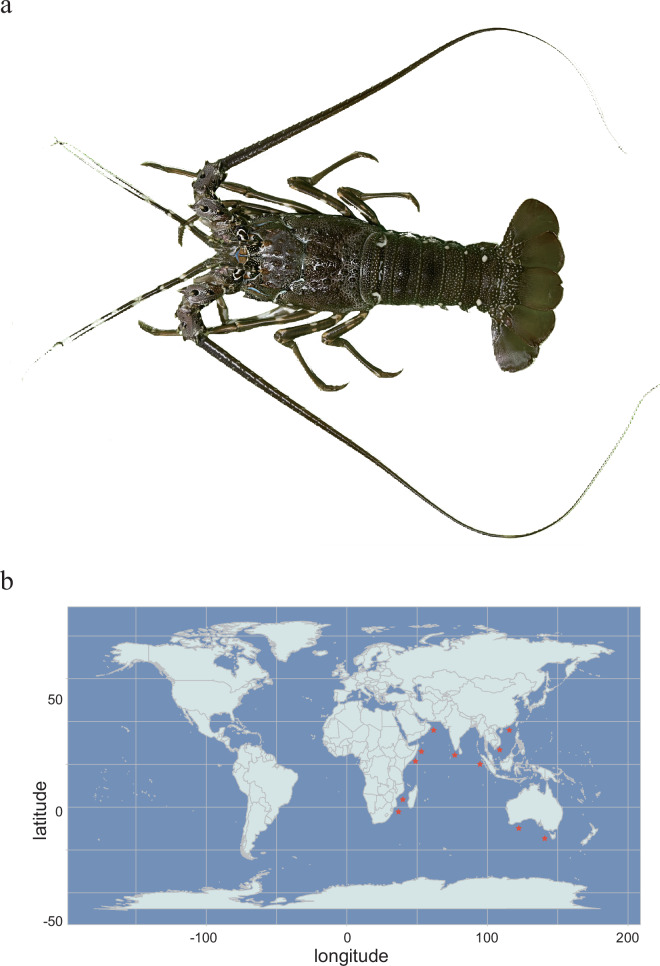
Photograph and geographic distribution of the long-tailed marine-living scalloped spiny lobster, *P. homarus*. (**a**) Photograph of an adult *P. h. homarus*. (**b**) Natural distribution map of *P. homarus*, indicated by the red star.

The scalloped spiny lobster, *Panulirus homarus*, belongs to Crustacea, Decapoda, Palinuridae, and *Panulirus*, and it consists of three economically valuable subspecies, including *Panulirus h. homarus* (Fig. 1a), *Panulirus h. megasculptus*, and *Panulirus h. rubellus*^1,2^. *P. h. rubellus* has also been reported to be a distinct species^3,4^. The species is an “engineer species” in coral reefs and rocky ecosystems, controlling population size by preying on benthic invertebrates (e.g., sea urchins, shellfish), maintaining ecological balance, and preventing overpopulation of certain species, which could destroy habitat structure^5^. In addition, *P. homarus* is distributed widely in the Indo-West Pacific (Fig. 1b), providing a good model for genetic comparisons of different geographic populations and for resolving gene flow, species differentiation, and ecological adaptation mechanisms^4,6^.

With increasing pressure on marine ecosystems globally, aquaculture is increasingly recognized as an important strategy for mitigating the depletion of wild fisheries resources^7^. *P. homarus* exhibits more rapid growth than other lobsters and it has been successfully farmed on a large scale in Vietnam and Indonesia, making it the optimal candidate for intensive large-scale lobster aquaculture^8,9^. Currently, the artificial nursery technology for scalloped spiny lobster remains valuable, with larvae mainly captured from the wild and then farmed in cage or industrial recirculating aquaculture systems^9,10^. Aquaculture research on *P. homarus* covers a range of topics, including resource assessment^9^ and the effects of nutrition^11,12^, salinity^13^, light^14^, and temperature^15^ on growth and reproduction. In addition, high-throughput sequencing technologies have advanced the study of lobster species genomes.

Currently, only the genomes of *Homarus americanus*^16^ and *P. ornatus*^17^ have been reported successfully. Genome assembly data for 13 *Panulirus* species are available in the NCBI database (Table S1). However, the average genome size of the species is only 1.5 Gb, which is significantly smaller than the anticipated genome size for lobsters. For instance, the genome size of *P. h. homarus* is merely 1.3 Gb, with a mere scaffold N50 of 2.9 kb, rendering the assembly quality inadequate to meet the demands of further research (Table S1). A comprehensive understanding of *P. homarus* is essential for effective management of its resources and the development of sustainable aquaculture practices.

**Fig. 2:**
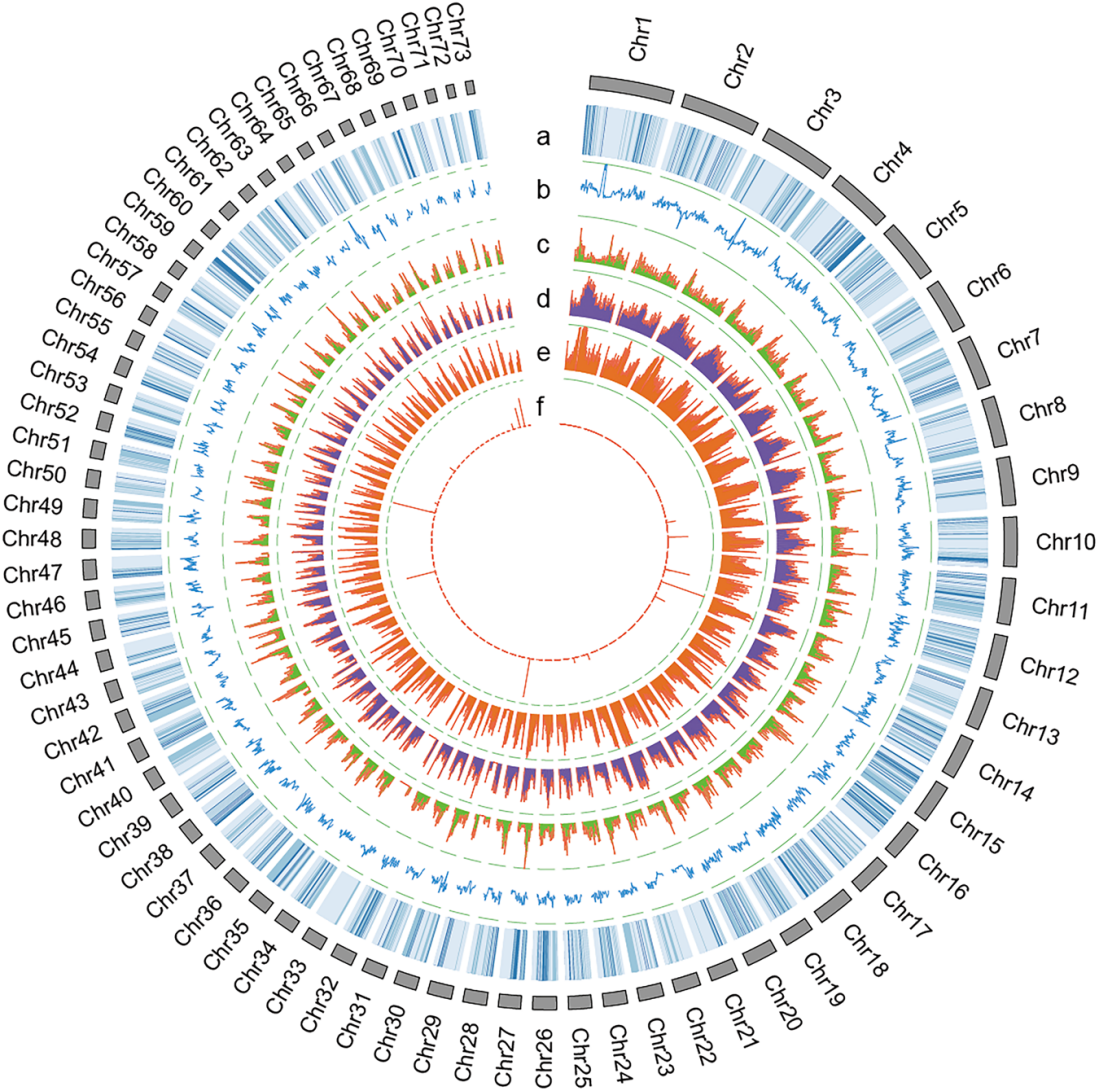
Genomic landscape of *P. h. homarus*. Circos plot illustrating the genomic features of *P. h. homarus*. From the outermost to innermost rings: (**a**) gene density, (**b**) GC content, (**c**) densities of DNA transposons, (**d**) density of Long Terminal Repeats (LTRs), (**e**) density of Long Interspersed Nuclear Elements (LINEs), and (**f**) density of Short Interspersed Nuclear Elements (SINEs), all represented in 200-kb genomic windows.

In the present study, the authors adopted a comprehensive multi-platform sequencing approach, combining Illumina short-read sequencing, PacBio long-read sequencing, and Hi-C chromosome conformation capture technologies to generate a chromosome-level genome assembly for *P. h. homarus* (Fig. 2). The project generated 140.56 Gb of Illumina short-read data, 341.51 Gb of PacBio long-read data, and 364.32 Gb of Hi-C data, culminating in a final assembled genome with a size of 2.61 Gb, a contig N50 of 5.43 Mb, and a scaffold N50 of 36.69 Mb (Tables 1, 2). The chromosome-level assembly enhances the genomic resources available for lobsters substantially and provides a crucial reference genome.

**Table 1:** Statistics of the sequencing data.

Library Types	Platform	Sample	Data size (Gb)	Average length (bp)	Sequencing Coverage (×)
WGS short reads	Illumina HiSeq 6000	Muscle	140.56	150	44.94
WGS long reads	Pacbio Sequel II	Muscle	341.51	—	109.19
Hi-C	Illumina HiSeq 6000	Muscle	364.32	150	140
RNA-seq	Illumina HiSeq 6000	Eye stalk, Hemocyte, Liver, Muscle, Intestine and Gills	55.7	150	21

**Table 2:** Assembly statistics of the *P. h. homarus*.

	PacBio				Hi-C			
	Length		Number		Length		Number	
	Contig (bp)	Scaffold (bp)	Contig	Scaffold	Contig (bp)	Scaffold (bp)	Contig	Scaffold
Total	2,720,451,987	2,720,451,987	7,135	7,135	2,720,451,987	2,720,451,987	7,145	4,288
Max	26,304,847	26,304,847	—	—	26,304,847	112,639,961	–	–
Number ≥ 100 Kb	2,580,191,777	2,580,191,777	1,551	1,551	2,580,111,777	2,636,992,862	1,556	203
N50	5,465,584	5,465,584	127	127	5,430,566	36,693,459	129	21
N90	353,615	353,615	842	842	350,563	18,068,482	847	62

## Methods

### Sample collection

An adult male *P. h. homarus* specimen was sourced from Hainan Yonghe Biotechnology Co., Ltd. (Qionghai, Hainan, China). Muscle tissue was collected after the lobster was anesthetized using cryogenic methods. The surface of the tissue was washed thoroughly several times with sterile phosphate buffered saline to effectively remove bacteria and impurities. The extraction of genomic DNA (gDNA) from muscle tissue for genome survey and library construction was carried out using the AMPure bead cleanup kit (Beckman Coulter, High Wycombe, UK) in strict accordance with the manufacturer’s instructions.

Total RNA was isolated from eye stalk, hemocyte, liver, muscle, intestine, and gills of the same specimen using TRIzol reagent, according to the manufacturer’s protocol. The integrity and quality of the RNA were evaluated by 1.5% agarose gel electrophoresis, whereas the concentrations were quantified precisely using a Qubit fluorometer (Thermo Fisher Scientific, Waltham, MA, USA).

### Genome sequencing

A short-read library with an insert size of 350 bp was constructed and sequenced on the Illumina Novaseq-6000 (Illumina Inc., San Diego, CA, USA) platform, generating 2 × 150 bp paired-end reads. In total, 0.08 μg gDNA per sample was used as input material for the DNA library preparations. Library preparation was performed using the NEBNext® Ultra™ DNA Library Prep Kit (New England Biolabs, Ipswich, MA, USA), in strict accordance with the Illumina second-generation sequencing protocol, resulting in 140.56 Gb of raw data (Table 1).

For PacBio sequencing, gDNA was employed to construct SMRTbell libraries and sequenced on the PacBio Sequel (PacBio, Menlo Park, CA, USA) platform, leveraging single molecule real-time (SMRT) technology. In brief, the genomic DNA was first sheared into 6–20-kb fragments using g-TUBE. Subsequently, ExoVII (New England Biolabs, Beverly, MA, USA) was used to remove single-strand overhangs, followed by DNA damage repair with the SMRTbell Express Template Preparation Kit 2.0 (PacBio). T4 DNA polymerase and T4 PNK (New England Biolabs) were used to repair the ends, making them suitable for ligating SMRTbell hairpin adapters. After ligation, EXOIII (New England Biolabs) and ExoVII (New England Biolabs) enzymes were used to remove imperfect templates, and AMPure PB beads were used for purification. Subsequently, sequencing primers were annealed to the SMRTbell templates, and polymerase was bound to the template ends using the Binding Kit (PacBio). Finally, the library was loaded onto SMRT Cells for sequencing. A total of 341.51 Gb of continuous long reads was obtained, resulting in an extensive 131-fold coverage of the *P. h. homarus* genome (Table 1).

For Hi-C sequencing, high molecular weight gDNA was first cross-linked and then digested using the MboI restriction enzyme (New England Biolabs). The DNA was mechanically sheared into 300–500-bp fragments following 5′ overhang biotinylation and blunt-end ligation. Finally, the Hi-C library was sequenced on the Illumina NovaSeq 6000 platform (lllumina Inc., San Diego, CA, USA) in a 2 × 150-bp paired-end strategy, yielding 364.32 Gb raw reads, with a sequencing depth of 140× (Table 1).

For RNA sequencing, libraries of six tissues were prepared using the NEBNext® UltraTM RNA Library Prep Kit (New England Biolabs) for Illumina®, with all procedures following the manufacturer’s protocol rigorously. The RNA-seq libraries were subsequently sequenced on the Illumina NovaSeq 6000 platform in a 2 × 150-bp paired-end strategy and producing 55.7 Gb of clean reads.

### Genome survey and assembly strategy

Prior to genome assembly, adapter sequences and low-quality reads from short-read sequencing data were filtered using Fastp software (v0.23.1)^18^ with default parameters, ensuring that only high-quality clean reads were retained for downstream processes. A comprehensive genome survey was performed to ascertain essential genomic characteristics, including overall size, heterozygosity, and repeat content. This was achieved by analyzing 17 distinct K-mer frequencies using SOAPec (v2.01)^19^ and GenomeScope (v2.0)^20^. Based on the analyses, the genome size of *P. h. homarus* was estimated to be 3,127.74 Mb, with 1.04% heterozygosity and 66.75% repetitive sequences at the dominant peak depth of 26 (Table S2, Fig. S1).

A dual-strategy utilizing two independent assembly software—Wtdbg2 (v2.5)^21^ and Flye (v2.9)^22^ with default parameters was employed for *P. h. homarus* genome assembly. The assembled genome drafts were subsequently improved using the Arrow (v8.0) polishing process^23^. The assembly results generated by Wtdbg2 and Flye after initial polish were merged using Quickmerge (v0.3)^24^. The merged assembly underwent further refinement through a comprehensive polishing process, involving two rounds of Arrow polishing followed by two rounds of Pilon polishing (v1.22)^25^, both utilizing default parameters. PacBio subreads were employed for Arrow polishing, whereas Illumina short reads were used for Pilon polishing to ensure high sequence accuracy. The rigorous and iterative assembly process culminated in the generation of 7,135 contigs, and a total assembly length of 2,720,451,987 bp (Table 2), representing a high-quality and robust genome assembly for *P. h. homarus*.

### Chromosome-level assembly refinement

**Fig. 3:**
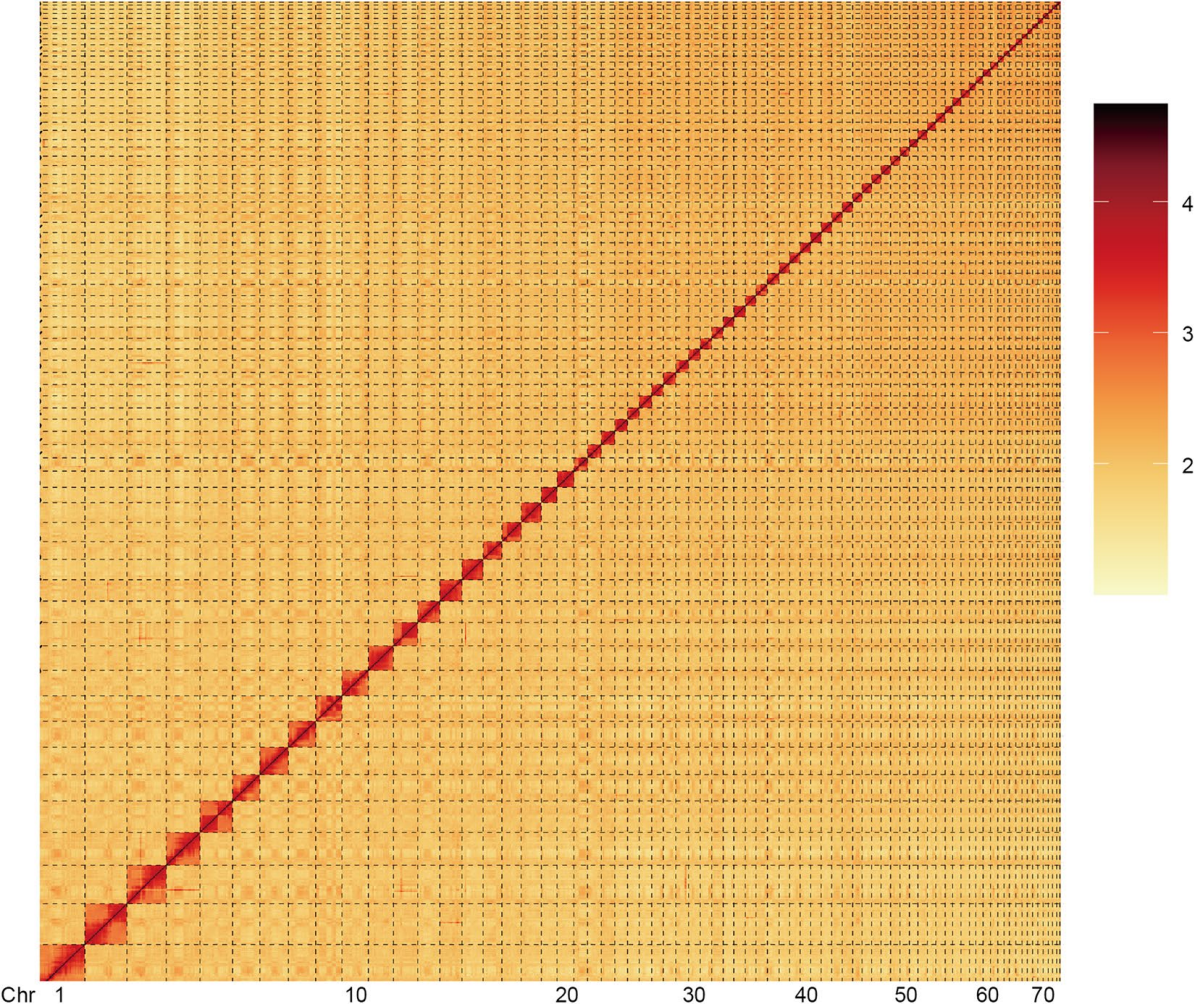
Hi-C heatmap (200-kb resolution) displaying interaction frequencies between different chromosomes of the *P. h. homarus*.

During the Hi-C scaffolding phase, the Juicer pipeline^26^ was used to align Fastp (v0.23.1)^18^ processed high-quality reads with draft genome assembly. The alignment was followed by the assembly of contigs into chromosomes, as well as to orient and sort contigs within each chromosome using the 3D-DNA pipeline^27^. Further refinement of the assembly was achieved through manual error correction using Juicebox Assembly Tools (v2.13.06)^26^. The rigorous scaffolding process successfully anchored 2,613.14 Mb of the genome to 73 chromosomes (Fig. 3), representing 96.05% of the total genome assembly (Table S3). The final assembly achieved a scaffold N50 of 36.69 Mb, reflecting a high level of continuity (Table 2). Remarkably, this assembly demonstrates exceptional contiguity, with 37 chromosomes containing no more than 30 gaps each (Table 3).

**Table 3:** Assembly statistics for chromosomes.

Name	Length (bp)	Gaps	Name	Length (bp)	Gaps
Chromosome 1	112,639,961	179	Chromosome 38	27,782,545	47
Chromosome 2	104,290,116	77	Chromosome 39	28,438,183	19
Chromosome 3	97,843,864	118	Chromosome 40	26,697,122	21
Chromosome 4	80,935,635	47	Chromosome 41	28,449,541	26
Chromosome 5	79,590,108	68	Chromosome 42	27,426,116	23
Chromosome 6	69,082,140	82	Chromosome 43	27,157,003	100
Chromosome 7	69,899,624	65	Chromosome 44	26,518,240	13
Chromosome 8	66,932,414	69	Chromosome 45	26,132,569	29
Chromosome 9	64,945,682	118	Chromosome 46	24,560,252	25
Chromosome 10	66,065,530	43	Chromosome 47	26,105,424	35
Chromosome 11	62,108,258	32	Chromosome 48	25,038,485	25
Chromosome 12	59,498,484	108	Chromosome 49	24,973,188	11
Chromosome 13	54,591,468	68	Chromosome 50	23,806,261	72
Chromosome 14	56,095,917	27	Chromosome 51	23,211,108	17
Chromosome 15	52,495,653	30	Chromosome 52	24,201,984	25
Chromosome 16	47,785,461	60	Chromosome 53	22,596,194	22
Chromosome 17	48,437,508	39	Chromosome 54	24,225,092	25
Chromosome 18	50,027,555	38	Chromosome 55	22,245,079	21
Chromosome 19	40,000,573	45	Chromosome 56	21,614,847	31
Chromosome 20	42,170,432	9	Chromosome 57	21,046,112	43
Chromosome 21	36,693,459	80	Chromosome 58	19,447,850	14
Chromosome 22	35,037,846	44	Chromosome 59	19,825,008	7
Chromosome 23	33,536,590	30	Chromosome 60	19,548,030	8
Chromosome 24	32,079,499	31	Chromosome 61	19,478,725	61
Chromosome 25	31,293,874	8	Chromosome 62	18,068,482	29
Chromosome 26	32,181,948	16	Chromosome 63	16,043,536	22
Chromosome 27	30,149,904	46	Chromosome 64	16,542,844	13
Chromosome 28	31,795,294	44	Chromosome 65	16,580,721	24
Chromosome 29	32,702,202	28	Chromosome 66	16,194,237	58
Chromosome 30	30,606,077	41	Chromosome 67	15,622,217	14
Chromosome 31	30,012,299	37	Chromosome 68	15,038,167	35
Chromosome 32	29,833,371	40	Chromosome 69	14,624,864	54
Chromosome 33	28,546,363	17	Chromosome 70	14,610,066	6
Chromosome 34	28,114,090	7	Chromosome 71	12,996,680	18
Chromosome 35	27,926,084	27	Chromosome 72	10,502,822	24
Chromosome 36	29,289,122	16	Chromosome 73	10,503,264	18
Chromosome 37	30,048,963	61			

### Comprehensive annotation of repetitive elements and noncoding RNAs

**Fig. 4:**
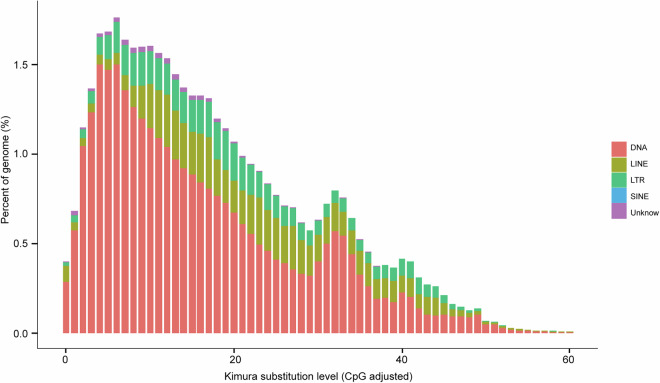
Distribution of divergence rates for transposable elements (TEs) in the *P. h. homarus* genome.

Repetitive sequences in the *P. h. homarus* genome were predicted using two strategies, including *de novo* assembly and homology matching^28^. Initially, the de novo-predicted repetitive sequence database was integrated with the Repbase homologous repetitive sequence database^29^. A suite of tools—including RepeatScout (v1.0.5)^28^, RepeatModeler (v2.0.1)^30^, Piler (v1.0)^31^, and LTR-FINDER (v1.0.6)^32^—was employed to identify transposable element (TE) families. Subsequently, classification of distinct repetitive elements was performed using RepeatMasker (v4.1.0)^30^, RepeatProteinMask (v4.1.0), and TRF (v4.0.9)^33^, aligning the *P. h. homarus* genome sequences against the integrated repetitive sequence database. After removing redundant entries from the various methods, repetitive sequences were observed to constitute 69.67% of the *P. h. homarus* genome (Table S4). Additionally, the Kimura divergence values of TEs were calculated using the ‘calcDivergenceFromalign.pl’ script^34^, and TE landscapes were visualized with ‘createRepeatLandscape.pl’^35^. The repeat elements identified included DNA transposons, which comprised 9.82% of the genome, long interspersed nuclear elements accounting for 37.44%, short interspersed nuclear elements representing 0.02%, and long terminal repeats making up 30.06% (Table 4 and Fig. 4).

**Table 4:** Classification of repetitive sequences in the *P. h. homarus* genome.

Type	Denovo + Repbase		TE Proteins		Combined TEs	
	Length (bp)	% of Genome	Length (bp)	% of Genome	Length (bp)	% of Genome
DNA	255,660,900	9.4	13,576,708	0.5	267,245,274	9.82
LINE	747,359,181	27.47	557,983,621	20.51	1,018,618,779	37.44
SINE	457,003	0.02	—	—	457,003	0.02
LTR	811,059,942	29.81	12,735,819	0.47	817,820,561	30.06
Other	—	—	—	—	—	—
Unknown	28,328,468	1.04	—	—	28,328,468	1.04
Total	1,661,445,638	61.07	583,800,643	21.46	1678379094	61.69

Tools specialized for non-coding RNAs (ncRNAs) were used to annotate ncRNA in *P. h. homarus* genome. tRNA were identified using tRNAScan (v1.4)^36^, whereas rRNA were predicated via BLAST (v2.2.26)^37^. Additional ncRNAs, including miRNAs and snRNAs, were annotated by aligning sequences with the Rfam database^38^ using the INFERNAL tool (v1.0)^39^. Ultimately, four distinct classes of ncRNAs were annotated successfully, comprising 20,765 miRNAs, 3,608 tRNAs, 1,421 rRNAs, and 3,066 snRNAs (Table 5), providing valuable insight into the non-coding RNA landscape of *P. h. homarus*.

**Table 5:** Classification of non-coding RNAs in the *P. h. homarus* genome.

nc RNA type		Copy (w*)	Average length (bp)	Total length (bp)	Proportion in Genome (%)
miRNA		20,765	176.05	3,655,685	0.13
tRNA		3,608	74	266,988	0.009814
rRNA	rRNA	1,421	168.66	239,664	0.00881
	18S	1,169	183.93	215,009	0.007903
	28S	13	131.92	1,715	0.000063
	5.8S	0	0	0	0
	5S	239	95.98	22,940	0.000843
snRNA	snRNA	3,066	219.44	67,2801	0.024731
	CD-box	2,434	206.85	50,3467	0.018507
	HACA-box	419	314.63	131,832	0.004846
	splicing	123	146.29	17,994	0.000661

### Integrated gene structure prediction and functional annotation

**Fig. 5:**
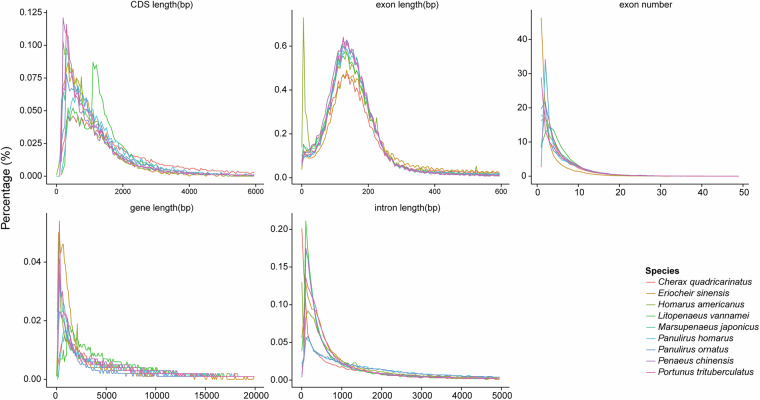
Comparisons of genomic elements across closely related species.

A comprehensive, integrated approach combining ab initio prediction, homology prediction, and transcriptome sequencing-based prediction was used to predict the gene structure of the *P. h. homarus* genome. For *de novo* gene predication, a robust suite of tools—AUGUSTUS (v3.2.3)^40^, GlimmerHMM (v3.02)^41^, SNAP (v2013.11.29)^42^, Geneid (v1.4)^43^, and Genscan (v1.0)^44^—was employed to predict gene structures directly from the genome sequence. For homology-based annotation, protein sequences from *Drosophila melanogaster* (fruit fly), *Penaeus chinensis* (Chinese shrimp), *Eriocheir sinensis* (Chinese mitten crab), *Litopenaeus vannamei* (Pacific white shrimp), *Marsupenaeus japonicus* (Kuruma shrimp), *P. ornatus* (ornate spiny lobster), *Portunus trituberculatus* (swimming crab), and *H. americanus* (American lobster) were retrieved from NCBI’s GenBank database and aligned to the *P. h. homarus* genome using BLAST (v2.2.26)^37^ and Genewise (v2.4.1)^45^. The integrated multifaceted strategy enabled comprehensive and accurate prediction of protein-coding genes, significantly advancing our understanding of the genetic architecture of *P. h. homarus*. A total of 8,545–178,660 homologous genes were identified for *D. melanogaster*, *P. chinensis*, *E. sinensis*, *L. vannamei*, *M. japonicus*, *P. ornatus*, *P. trituberculatus*, and *H. americanus* (Table 6). Gene length, along with CDS, exon, and intron lengths, was analyzed and compared to those of other species (Fig. 5). The mean transcript length in *P. h. homarus* was 31,472.77 bp, with CDS, exon, and intron lengths averaging 1,613.73 bp, 279.37 bp, and 6,251.44 bp, respectively (Table S5).

**Table 6:** Statistical analyses of gene structure annotation of the *P. h. homarus* genome.

	Gene set	Number	Average transcript length (bp)	Average CDS length (bp)	Average exons/gene	Average exon length (bp)	Average intron length (bp)
*De novo*	Augustus	659,318	1,799.42	355.72	1.73	205.12	1,966.25
	GlimmerHMM	132,893	8,676.42	796.74	2.51	317.35	5,216.16
	SNAP	875,253	2,584.61	394.55	2.05	192.48	2,086.10
	Geneid	267,413	6,799.47	519.05	2.37	219.04	4,585.32
	Genscan	79,061	21,323.13	1,286.12	5.23	246.05	4,740.20
Homolog	*D. melanogaster*	8,545	14,666.23	979.91	3.9	251.47	4,724.85
	*E. sinensis*	84,894	3,107.67	723.74	1.64	441.67	3,732.89
	*H. americanus*	135,558	2,773.94	678.41	1.6	424.38	3,500.81
	*L. vannamei*	70,184	3,806.52	946.38	1.92	492.93	3,109.10
	*M. japonicus*	75,131	5,650.06	796.72	2.08	383.73	4,509.48
	*P. chinensis*	178,660	2,395.58	562.63	1.48	379.61	3,801.87
	*P. ornatus*	96,200	3,829.82	616.76	1.97	312.78	3,306.05
	*P. trituberculatus*	37,464	8,318.17	791.42	2.66	297.57	4,535.37
RNAseq	PASA	59,275	55,389.17	4,422.12	7.22	612.15	8,188.90
	Cufflinks	118,883	11,285.66	856.62	2.77	309.37	5,895.81
EVM		118,883	11,285.66	856.62	2.77	309.37	5,895.81
Pasa-update		118,774	11,433.75	858.88	2.77	309.67	5,962.76
Final set		25,580	31,472.77	1,613.73	5.78	279.37	6,251.44

*De novo*: Gene structure predictions performed using Augustus, GlimmerHMM, SNAP, Geneid, and Genscan software.

Homolog: Gene annotations identified through comparison with orthologs from related species.

RNA-seq: Gene structures annotated based on transcriptome sequencing data.

PASA: Gene structures obtained from the assembly of RNA-seq transcripts using Trinity and integrated with transcriptome data.

Pasa-update: Corrections to gene structures based on PASA annotations.

EVM: Integrated gene annotations combining *de novo*, homologous, and RNA-seq annotation results using EvidenceModeler (EVM).

Final set: The finalized set of functionally annotated genes after integrating and refining all annotation methods.

Transcript numbers indicate the identified transcript variants from each annotation strategy.

Average transcript length, CDS length, exon number, and exon/intron lengths are based on the corresponding gene prediction methods listed.

**Fig. 6:**
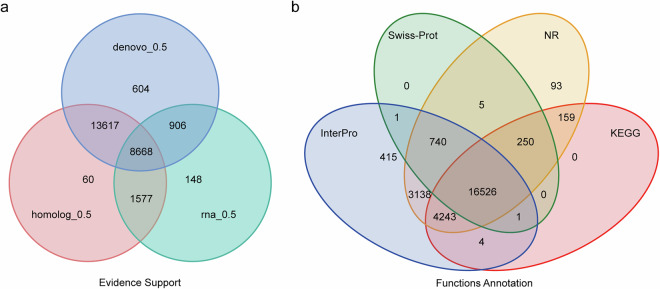
Gene prediction and functional annotation of the *P. h. homarus* genome. (**a**) Venn diagram illustrating the integration of gene set predictions from various methods. (**b**) Venn diagram showing overlap of functional annotations based on different databases.

To further refine transcriptome assembly, two distinct methods were employed: genome-guided transcript assembly and de novo assembly using Trinity software (v2.11.0)^46^. Gene structures were identified using PASA (v2.1.0)^47^, and gene sets predicted through various methods were integrated into a non-redundant comprehensive gene set of 25,580 protein-coding genes using Evidence Modeler (v1.1.1)^48^ (Table 7 and Fig. 6a).

**Table 7:** Summary of functional gene annotation in the *P. h. homarus* genome.

	Number	Percent (%)
Total	25,580	—
Swiss-Prot	17,523	68.5
Nr	25,154	98.3
KEGG	21,183	82.8
InterPro	25,068	98
GO	21,997	86
Pfam	11,490	44.9
Annotated	25,575	100
Unannotated	5	0.0

Functional annotation of these protein-coding genes was performed using BLASTp (v2.2.26)^37^ and Diamond (v0.8.22)^49^ to align sequences against several key protein databases, including SwissProt^50^, NCBI Nonredundant protein (NR), KEGG^51^, InterPro^52^, Gene Ontology (GO)^53^, and Pfam^54^, with an E-value cutoff of 1E-5. Protein domains and motifs were annotated using InterProScan (v5.52–86.0)^55^. Among the 25,580 predicted genes, 25,575 (99.98%) were annotated to at least one database (Table 7) and 16,526 proteins (64.61%) received annotation support from across all four databases (Fig. 6b).

## Data Records

We have deposited the Hi-C sequencing data (SRR30872734), Illumina sequencing data (SRR30872735), PacBio sequencing data (SRR30872736), and transcriptomic sequencing data (SRR3105790260 - SRR3105790765) in the SRA at NCBI^56^.

The genome-wide shotgun project has been deposited in DDBJ/ENA/GenBank under accession number GCA_043589495.1^57^, and the genome assembly along with its annotation information has been made available on Figshare^58^.

## Technical Validation

The quality of the *P. h. homarus* genome assembly was technically verified rigorously through a multifaceted evaluation. First, the genomic quality was analyzed using Benchmarking Universal Single-Copy Orthologs (BUSCO) (v5.8.0)^59^, with the arthropoda_odb12 BUSCO database, to assess the presence of single-copy orthologous genes. Using tools such as tBLASTn (v2.2.26)^37^, AUGUSTUS (v3.2.3)^40^, and HMMER^60^, 98.2% of gene orthologs were detected, of which 97.2% were complete and 1.0% fragmented, indicating a highly comprehensive assembly (Table S6). Second, using the Core Eukaryotic Genes Mapping Approach (CEGMA) (v2.5)^61^, we identified homologs for 226 highly conserved core genes in *P. h. homarus*, representing 92.34% (229) of the total, further supporting the completeness of the assembly (Table S7). Third, the consensus quality value and k-mer (k = 21) completeness of the assembly evaluated using Merqury software^62^ were 31.78 and 87.59%, respectively (Table S8). In addition, alignment of Illumina sequencing reads to the nuclear genome using BWA (v0.7.8)^63^ yielded a high read mapping rate of 98.60% and a coverage rate of 94.85%, underscoring the robust integrity of the assembled genome and the consistency of the sequencing data (Table S9). Finally, to conduct genome-wide homology analysis, we used MCScanX within the JCVI toolkit (v1.1.12) (https://github.com/tanghaibao/jcvi) to perform a synteny comparison between the genomes of the *P. h. homarus* and *P. ornatus*, and visualized the macro-syntenic relationships using Circos (v0.69)^64^. The results showed that 73 chromosome-level scaffolds of *P. h. homarus* exhibited significant synteny with the corresponding chromosomes of *P. ornatus* (Fig. S2). These combined results affirm the exceptional quality and completeness of the *P. h. homarus* genome assembly.

## Supplementary information

Supplementary figure and table Legends

## Data Availability

In the present study, no custom code was developed. All commands and pipelines used for data processing are detailed comprehensively in the methods section. For software where specific parameters are not explicitly mentioned, we adhered to the default settings as recommended by the software developers. The core code is available at https://github.com/sundongfang/Genome-Assembly-of-Panulirus-homarus.
