# Supplementary material for: Chromosome-level genome assembly of scalloped spiny lobster Panulirus homarus homarus
Source: Sci Data. 2025 May 28;12:900. doi: 10.1038/s41597-025-05253-9 (PMC12120130; doi:10.1038/s41597-025-05253-9)
Supplement: Supplementary file 1 — Supplementary figure and table Legends [file 41597_2025_5253_MOESM1_ESM.pdf]

Supplementary figure and table Legends

Fig. S1 Estimated genome size of *P. h. homarus* based on K-mer analysis.

Fig. S2 Chromosome sequence synteny comparisons. Syntenic relationship between the *P. h. homarus* genome and the *P. ornatus* genome.

Table S1. Genome statistics of 13 *Panulirus* species in NCBI.

Table S2. Statistics of genome survey based on 17-mer analysis.

Table S3. Statistics of genome mount rate of *P. h. homarus*.

Table S4. Repetitive sequence statistics results.

Table S5. Basic statistical results of gene structure of nearby species.

Table S6. BUSCO assessment results.

Table S7. Genome CEGMA evaluation results.

Table S8. Statistics of assembly quality and completeness evaluation using Merqury.

Table S9. The genome coverage statistics of Illumina reads.

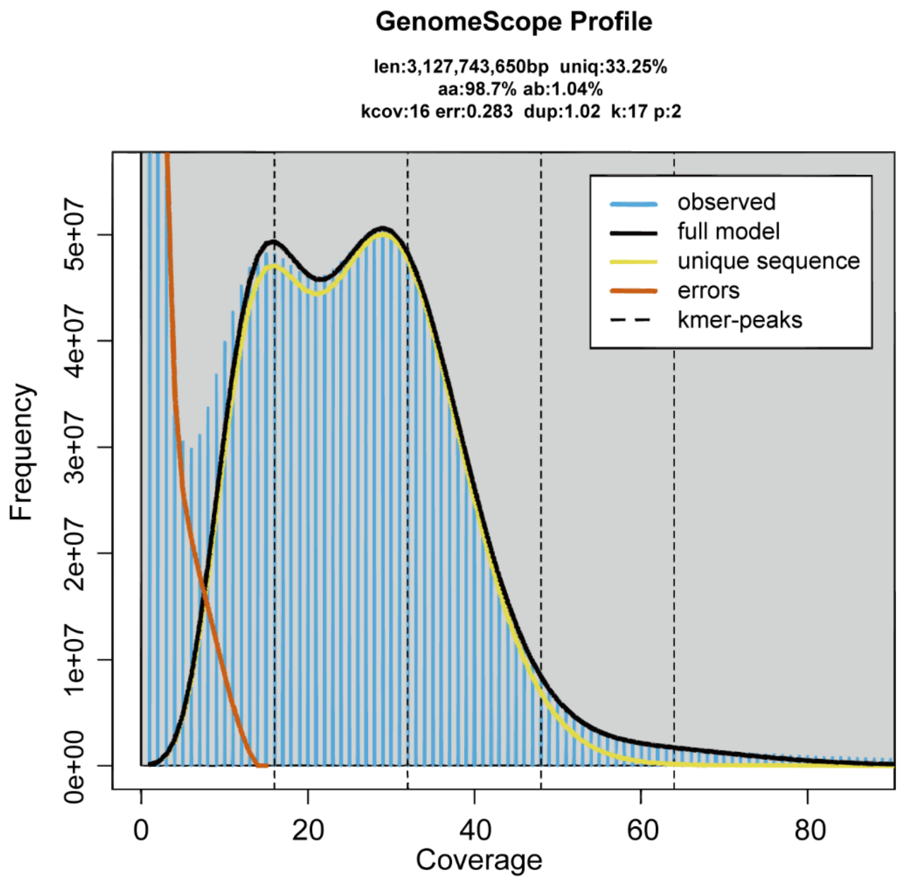

Fig. S1 Estimated genome size of *P. h. homarus* based on K-mer analysis.

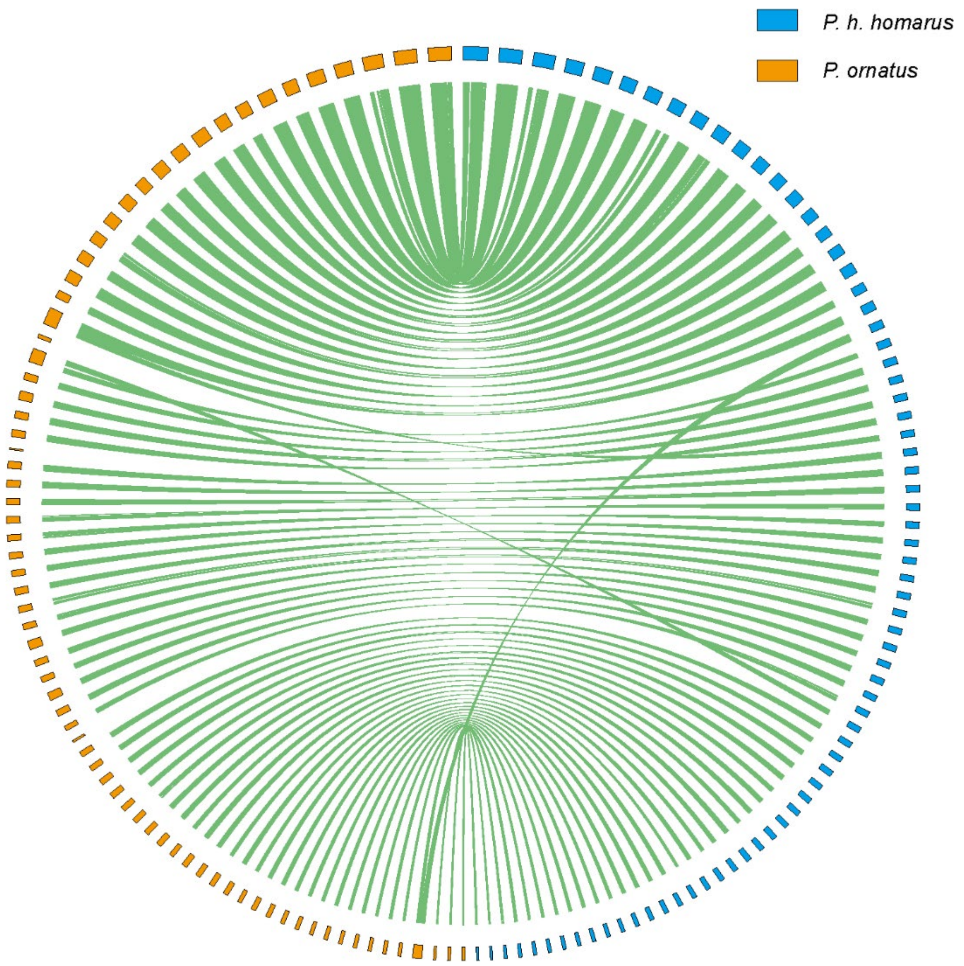

Fig. S2 Chromosome sequence synteny comparisons. Syntenic relationship between the *P. h. homarus* genome and the *P. ornatus* genome. Each line connects a pair of homologous sequences between the two species. Blue and orange colors represent the 73 chromosomes of *P. h. homarus* and *P. ornatus*, respectively**.**

Table S1 Genome statistics of 13 *Panulirus* species in NCBI.

GenBank numbers	Specises name	Genome size	Total ungapped length	Number of chromosomes	Number of scaffolds	Scaffold N50	Scaffold L50	Number of contigs	Contig N50	Contig L50	GC percent	Genome coverage	Assembly level
GCA_036320965.1	*Panulirus ornatus*	2.7 Gb	2.7 Gb	73	1,450	51 Mb	20	8058	5.1 Mb	132	43	188.0x	Chromosome
GCA_038088735.1	*Panulirus argus*	1.8 Gb	1.8 Gb	/	424,852	6.9 kb	72,611	434,038	6.8 kb	75,888	42.5	90.0x	Scaffold
GCA_032361405.1	*Panulirus homarus*	1.3 Gb	1.3 Gb	/	618,289	2.9 kb	121,034	704,650	2.6 kb	140,428	42.5	50.0x	Scaffold
GCA_032273725.1	*Panulirus interruptus*	1.4 Gb	1.4 Gb	/	748,487	2.5 kb	161,943	859,704	2.2 kb	187,099	42.5	50.0x	Scaffold
GCA_032361705.1	*Panulirus versicolor*	1.5 Gb	1.5 Gb	/	466,583	4.2 kb	94,215	518,312	3.9 kb	105,690	43	50.0x	Scaffold
GCA_032273845.1	*Panulirus longipes*	1.2 Gb	1.2 Gb	/	852,228	1.8 kb	217,975	972,289	1.6 kb	245,603	43	50.0x	Scaffold
GCA_032361485.1	*Panulirus cygnus*	0.94 Gb	0.94 Gb	/	844,337	1.2 kb	247,865	926,678	1.1 kb	267,453	43	50.0x	Scaffold
GCA_032361385.1	*Panulirus guttatus*	1.6 Gb	1.6 Gb	/	657,369	3.6 kb	128,177	746,306	3.2 kb	147,559	43	50.0x	Scaffold
GCA_032273605.1	*Panulirus laevicauda*	1.4 Gb	1.4 Gb	/	552,489	3.5 kb	106,893	618,989	3.2 kb	122,167	43	50.0x	Scaffold
GCA_032361765.1	*Panulirus inflatus*	1.3 Gb	1.3 Gb	/	632,377	2.9 kb	127,289	705,867	2.6 kb	143,870	42.5	50.0x	Scaffold
GCA_032361445.1	*Panulirus gracilis*	1.3 Gb	1.3 Gb	/	679,949	2.5 kb	148,520	751,506	2.3 kb	166,128	42.5	50.0x	Scaffold
GCA_032361885.1	*Panulirus marginatus*	1.3 Gb	1.3 Gb	/	842,519	1.8 kb	210,730	959,315	1.7 kb	238,259	42.5	50.0x	Scaffold
GCA_032361865.1	*Panulirus pascuensis*	1.1 Gb	1.1 Gb	/	848,215	1.6 kb	223,628	989,875	1.4 kb	256,022	43	50.0x	Scaffold
GCA_018397875.1	*Panulirus ornatus*	1.9 Gb	1.8 Gb	/	403,881	8.1 kb	35,127	562,448	5.4 kb	81,259	42.5	40.0x	Scaffold
GCA_032273525.1	*Panulirus ornatus*	1.4 Gb	1.4 Gb	/	666,649	2.7 kb	144,629	739,006	2.5 kb	162,785	43	50.0x	Scaffold

Table S2. Statistics of genome survey based on 17-mer analysis

K-mer number	K-mer Depth (X)	Genome Size (Mbp)	Heterozygous Ratio (%)	Repeat (%)
1,127,917,413	26	3,127.74	1.04	66.75

Table S3. Statistics of genome mount rate of *P. h.homarus*

Class	Scaffold Number	Total Length
place	73	2,613,136,226 bp
unplace	4,215	107601461 bp
total	4,288	2,720,737,687 bp
Genome mount rate	96.05%	

Table S4. Repetitive sequence statistics results.

Type	Repeat Size(bp)	% of genome
Trf	610,986,788	22.46
Repeatmasker	1,661,445,638	61.07
Proteinmask	583,800,643	21.46
Total	1,895,226,719	69.67

Table S5. Basic Statistical Results of Gene Structure of Nearby Species.

Species	Number	Average transcript length (bp)	Average CDS length (bp)	Average exons per gene	Average exon length (bp)	Average intron length (bp)
Cherax quadricarinatus	44,570	25,129.54	2,416.93	5.5	439.7	5,050.92
Eriocheir sinensis	28,033	4,711.03	1,078.75	3.27	330.15	1,601.93
Homarus americanus	25,068	8,511.39	1,171.40	5.47	214.34	1,643.86
Litopenaeus vannamei	25,596	8,879.91	1,545.06	5.94	259.91	1,483.38
Marsupenaeus japonicus	24,317	12,027.62	1,237.45	5.5	224.79	2,395.18
Penaeus chinensis	25,024	11,290.42	1,230.06	5.88	209.23	2,061.94
Panulirus homarus homarus	25,580	31,472.77	1,613.73	5.78	279.37	6,251.44
Panulirus ornatus	22,752	29,857.91	1,420.49	5.51	257.65	6,300.84
Portunus trituberculatus	20,189	10,267.48	1,219.17	5.17	235.8	2,169.66

Table S6. BUSCO assessment results.

Species	BUSCO notation assessment results
P. h. homarus	C: 98.2%[S: 97.2%,D: 1.0%],F: 1.0%,M: 0.8%, n: 1667

C：Complete BUSCOs

S：Complete and single-copy BUSCOs

D：Complete Duplicated BUSCOs

F： Fragmented BUSCOs

M： Missing BUSCOs

n： Total BUSCO groups searched

Table S7. Genome CEGMA evaluation results.

Species	Complete		Complete + Partial	
	# Prots	% Completeness	# Prots	% Completeness
P. h. homarus	194	78.23	229	92.34

Table S8. Statistics of assembly quality and completeness evaluation using Merqury.

Quality value (QV)	Error rate	Completeness (%)
31.78	0.0006635	87.5949

Table S9. The genome coverage statistics of Illumina reads.

		% of Percentage
Reads	Mapping rate (%)	98.6
Genome	Average sequencing depth	30.91
	Coverage (%)	94.85
	Coverage at least 4X (%)	92.66
	Coverage at least 10X (%)	88.24
	Coverage at least 20X (%)	74.1
